# Development of a MediaPipe-based framework for biomechanical quantification of table tennis forehand strokes

**DOI:** 10.3389/fspor.2025.1635581

**Published:** 2025-08-15

**Authors:** Yuanyuan Lyu, Xiaoling Duan, Chen Yang, Qiang Ye

**Affiliations:** ^1^ School of Sport and Health Science, Nanjing Sport Institute, Nanjing, China; ^2^ School of Table Tennis and Badminton, Nanjing Sport Institute, Nanjing, China; ^3^ Information Affairs Office, Nanjing Sport Institute, Nanjing, China

**Keywords:** motion analysis, MediaPipe, table tennis, forehand stroke, biomechanics

## Abstract

**Introduction:**

This study aimed to quantify kinematic relationships across body segments during forehand strokes to provide interpretable metrics for single-camera based lightweight table tennis diagnostics.

**Methods:**

We analyzed 34 female players (aged 9.1–21.7 years) from provincial teams, recording a total of 340 strokes (10 per player). An SVM model was used to predict ball speed, after which 320 strokes (8–10 per player) were retained by removing outliers in ball speed. From MediaPipe position time series, we calculated velocity, angle and angular velocity time series, and extracted kinematic parameters from these time series, including range, mean/peak/impact values. Within-subject correlation coefficients (*r*_ws_) were calculated to identify key biomechanical parameters that contribute to the ball speed, while between-subject correlation coefficients (*r*_bs_) were used to detect the relationship between age/height and ball speed.

**Results:**

Ball speed increased with greater playing-side arm linear movement at the shoulder (*r*_ws_ = 0.51 to 0.63), elbow (*r*_ws_ = 0.63 to 0.70) and wrist (*r*_ws_ = 0.50 to 0.60), as well as with enhanced rotational motion at the playing-side upper arm (*r*_ws_ = 0.65 to 0.71), shoulder line (*r*_ws_ = 0.54 to 0.57), and hip line (*r*_ws_ = 0.51 to 0.59). Conversely, ball speed decreased with excessive contralateral shoulder horizontal flexion/extension (*r*_ws_ = −0.44 to −0.62) and playing-side elbow flexion-extension (*r*_ws_ = −0.35). At the population-level, ball speed increases with age before 14.3 years (*r*_bs_ = 0.68) but plateaus thereafter (*r*_bs_ = 0.17).

**Discussion:**

This MediaPipe-based framework demonstrates potential for efficient biomechanical analysis in table tennis, providing a promising foundation for lightweight real-time analysis solutions.

## 1 Introduction

Table tennis requires precise whole-body coordination and accurate stroke timing, creating unique biomechanical analysis challenges. Biomechanical analysis reveals underlying technique patterns and improves player training and performance. Researchers employ various devices to study motion characteristics: optical systems (1–5), pressure and force sensors (1, 2), electromyography (EMG) (3), and inertial measurement units (IMUs) attached to the body or racket (6).

Optical devices serve as primary tools, used alone or combined with other sensors. They capture precise three-dimensional spatial data while allowing players to move freely without interference. Researchers examine movement patterns across skill levels, identifying subtle joint and muscle dynamics invisible to the naked eye. Wang et al. used optical systems and EMG to compare elite and amateur players. Elite players showed greater ankle eversion and larger knee and hip flexion angles during backswing and follow-through phases (3). He et al. employed a VICON optical system to study driving leg kinematics during topspin forehand loops. They found significant ankle movement differences between elite and intermediate players, recommending that intermediate players enhance lower limb muscle response to improve energy transfer (7). Qian et al. compared superior and intermediate players using VICON, finding that superior players exhibited greater hip flexion and knee external rotation at stroke initiation, plus increased hip internal rotation and extension at stroke completion (1). However, these optical systems require controlled environments, expensive multi-camera setups, high-frequency capabilities, time-consuming marker placement, large laboratories, and skilled technicians. These limitations restrict widespread use in practical training.

Researchers have adopted lightweight machine learning-based pose estimation models (e.g., OpenPose, MediaPipe Pose, PoseNet, AlphaPose, DeepLabCut, HRNet, BlazePose, EfficientPose, MoveNet) as alternatives to complex optical motion analysis systems (8–12). These models provide human body landmarks for further development (8, 11). In sports and exercise, these models analyze movements (e.g., running, jumping, squatting) to optimize technique (8, 11, 12), detect injury-prone postures, and provide real-time form feedback via mobile apps (12). They also assess team dynamics, monitor exercise quality in fitness apps, and enable remote training guidance (9). Compared to other models requiring complex configurations, MediaPipe provides easy-to-use APIs and comprehensive documentation, lowering the development barrier. Developers can quickly integrate it into existing projects (9, 10, 13). These advantages make MediaPipe Pose the preferred solution suitable for scenarios requiring real-time performance and cross-platform deployment.

While lightweight machine learning-based pose estimation models exhibit lower precision compared to high-fidelity motion capture systems like VICON (10, 13, 14), recent advances in machine learning have significantly enhanced the utilization of keypoint data from lightweight pose estimation models (10, 15). Machine learning includes classical approaches and deep learning architectures. Integrating these methods with pose estimation data creates new opportunities for automated movement quality assessment (AQA) in athletic and rehabilitative applications.

Classical machine learning approaches include Decision Trees, Random Forest, SVM, Naive Bayes, K-NN, and Linear/Logistic Regression (9). These methods address classification (16–20), regression (21–23), clustering tasks, and feature importance ranking (24). Naive Bayes offers mathematical robustness and efficiency but relies on independence assumptions (16). Decision Trees are capable of identifying contributing factors in biomechanical analyses, with applications including knee biomechanical asymmetry (24). Random Forest combines multiple trees to improve prediction accuracy and reduce overfitting (24, 25), successfully predicting joint angles and moments (21). Linear regression predicts continuous outcomes, such as running stride temporal variables and peak vertical ground reaction force (22), while logistic regression addresses classification problems, such as binary musculoskeletal disorder classification (16, 17). Linear and logistic regression can work together to identify key predictors and examine high vs. low knee abduction moments (18). Support Vector Machines (SVM) provide nonlinear classification and regression capabilities through kernel methods, making them well-suited for complex biomechanical tasks among classical machine learning approaches. Applications of SVM include predicting athlete aerobic fitness (19), analyzing running gait (20), and predicting fastball speed using kinetic and kinematic predictors (23).

These classical machine learning approaches offer significant advantages in interpretability (16, 23) and computational efficiency (18, 21), making them well-suited for clinical applications and scenarios with limited data availability. However, their dependency on manual feature engineering (26), limited capacity for processing high-dimensional data structures (27), constrained nonlinear modeling capabilities, and insufficient handling of temporal sequences (28) restrict their effectiveness in advanced analytical tasks (26). In contrast, deep learning architectures—including Convolutional Neural Networks (CNNs), Recurrent Neural Networks/Long Short-Term Memory networks (RNNs/LSTMs), and Transformers—provide superior accuracy and automation capabilities for complex spacial or temporal movement analysis. Nevertheless, these approaches require substantial computational resources and large training datasets to achieve optimal performance (29).

CNNs excel at processing visual data through hierarchical structures and have been successfully applied to performance classification and kinetic parameter prediction (27, 30). While CNNs effectively extract spatial features, they struggle with temporal sequence modeling, prompting researchers to integrate CNNs with RNNs to achieve comprehensive spatial and temporal analysis (31, 32). RNNs handle time-series prediction effectively (29) but suffer from vanishing and exploding gradient problems (33). LSTM units address these limitations as a specialized RNN component (29, 33, 34). LSTMs have predicted joint reaction forces (35), segmented jump phases (36), and modeled stress evolution in skeletal muscle tissue (33). Transformers extend RNN capabilities by overcoming LSTM’s long-range dependency limitations. Their attention mechanisms capture relationships across entire sequences simultaneously, providing superior sequence modeling and interpretability through attention weights while enabling efficient parallelization.

Recent advances in table tennis research have leveraged lightweight machine learning models and pose estimation techniques to analyze player performance. Chen et al. enhanced swing recognition through an improved OpenPose framework integrated with MobileNet v3-small and InceptionTime architectures (8). Building on pose estimation approaches, Llanos et al. developed a comprehensive assessment system using OpenPose and SVM-RBF classifiers to differentiate four key postural elements: upper body lean, knee bend, forehand/backhand strokes, and footwork patterns (11). For real-time applications, He et al. combined YOLOv5 and MediaPipe for stroke and posture assessment, implementing dynamic time warping algorithms to compare temporal sequences of joint angles (elbow-shoulder-hip) between player motions and reference techniques (12).Similarly, Huang and colleagues employed OpenPose for skeleton extraction combined with SVM for real-time swing recognition, incorporating Dynamic Time Warping (DTW) algorithms to evaluate technique through keypoint trajectory comparison and identification of suboptimal joint movements (37). Transformer-based approaches have also shown promise, with Dong and colleagues utilizing MediaPipe for human keypoint extraction and employing Transformer models for stroke recognition across six distinct stroke types (38).

Despite advances in lightweight pose estimation for table tennis, existing methods focus primarily on stroke classification without quantifying how multi-segment body kinematics influence ball impact outcomes. These approaches have not yet established measurable relationships between whole-body movement patterns and performance metrics, limiting their utility for diagnostic applications in sports training.

This study aimed to quantify kinematic relationships across body segments during forehand strokes to provide interpretable metrics for lightweight table tennis diagnostics. Using single-camera whole-body landmark detection and ball speed measurement, we employed statistical analysis to identify key kinematic factors influencing ball performance. This research bridges lightweight human motion capture with actionable biomechanical insights for practical sports training applications.

## 2 Methods

The research method consists of the following steps: (1) Pre-train a Support Vector Machine (SVM) model to serve as a tool for direct ball speed measurement from video footage. (2) Participants perform forehand strokes while MediaPipe captures the 3D position series of human body landmarks. (3) Calibrate the position series to compute velocity, angle, and angular velocity sequences, from which kinematic summaries are extracted. (4) Apply the pre-trained SVM model to predict ball speed based on the ball trajectory. (5) Conduct statistical analysis to examine correlations between kinematic summaries and ball velocity, as well as relationships between participant demographics (age and height) and ball speed.

### 2.1 SVM ball speed model

A Support Vector Machine (SVM) regression model was pre-trained on ball coordinates (x and y values) extracted from video frames to predict ball speed. This model was then employed in the main experiment to streamline the measurement process, ensuring efficiency for practical applications.

Ball speed specifically denotes average horizontal ball speed, which critically measures offensive performance in table tennis. The table surface was divided into 10cm×10cm squares using fine lines to accurately identify ball landing spots and determine horizontal flight distance. A centrally positioned audio recorder captured sounds from ball-racket and ball-table contact. These sound waves were analyzed using Adobe Audition, which provides 1 millisecond temporal resolution, enabling precise measurement of impact time intervals. Corrections were applied based on sound velocity at 20°C (343 m/s) to account for varying sound travel times caused by different distances between impact points, drop points, and the recorder. Ball speed was calculated by dividing horizontal flight distance by travel time.

The SVM regression model was developed using 517 preliminary stroke measurements from standardized ball trajectories at controlled speeds. Manually annotated (x, y) coordinates from six consecutive video frames created 12-dimensional feature vectors capturing ball movement immediately before screen exit.

Using six-frame coordinates with SVM regression rather than simple two-point measurement offers several advantages: (1) Table tennis ball trajectories are inherently nonlinear due to air resistance, spin effects, and gravitational forces. SVM’s ability to find optimal decision boundaries in high-dimensional space makes it particularly suitable for processing multi-frame coordinate data and learning complex velocity patterns that simple kinematic equations cannot capture. (2) Six frames provide finer temporal sampling of ball motion, effectively filtering random noise and measurement errors common in single-frame coordinate detection. (3) If ball detection fails in one or two frames, SVM can still make accurate predictions using remaining frames, whereas two-point methods would fail completely.

**Figure 1:**
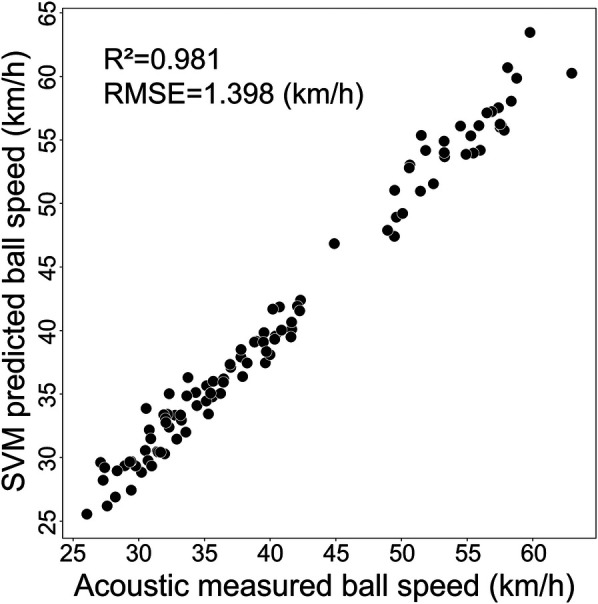
Validation of SVM ball speed prediction model against acoustic measurements with high accuracy.

The 517 preliminary measurements were split into training (80%) and test (20%) sets, with the test set remaining independent throughout the training process. Hyperparameter optimization employed 5-fold cross-validation with grid search across C parameters [0.1, 1, 10, 100, 1,000, 10,000, 100,000] and γ values [1, 0.1, 0.01, 0.001, 0.0001, 0.00001]. This strategy ensured robust parameter selection while preventing overfitting. The optimal configuration achieved C=100,000 and γ=0.001, yielding best cross-validation score (R^2^=0.987) and test set R^2^=0.981 (Figure 1).

**Figure 2:**
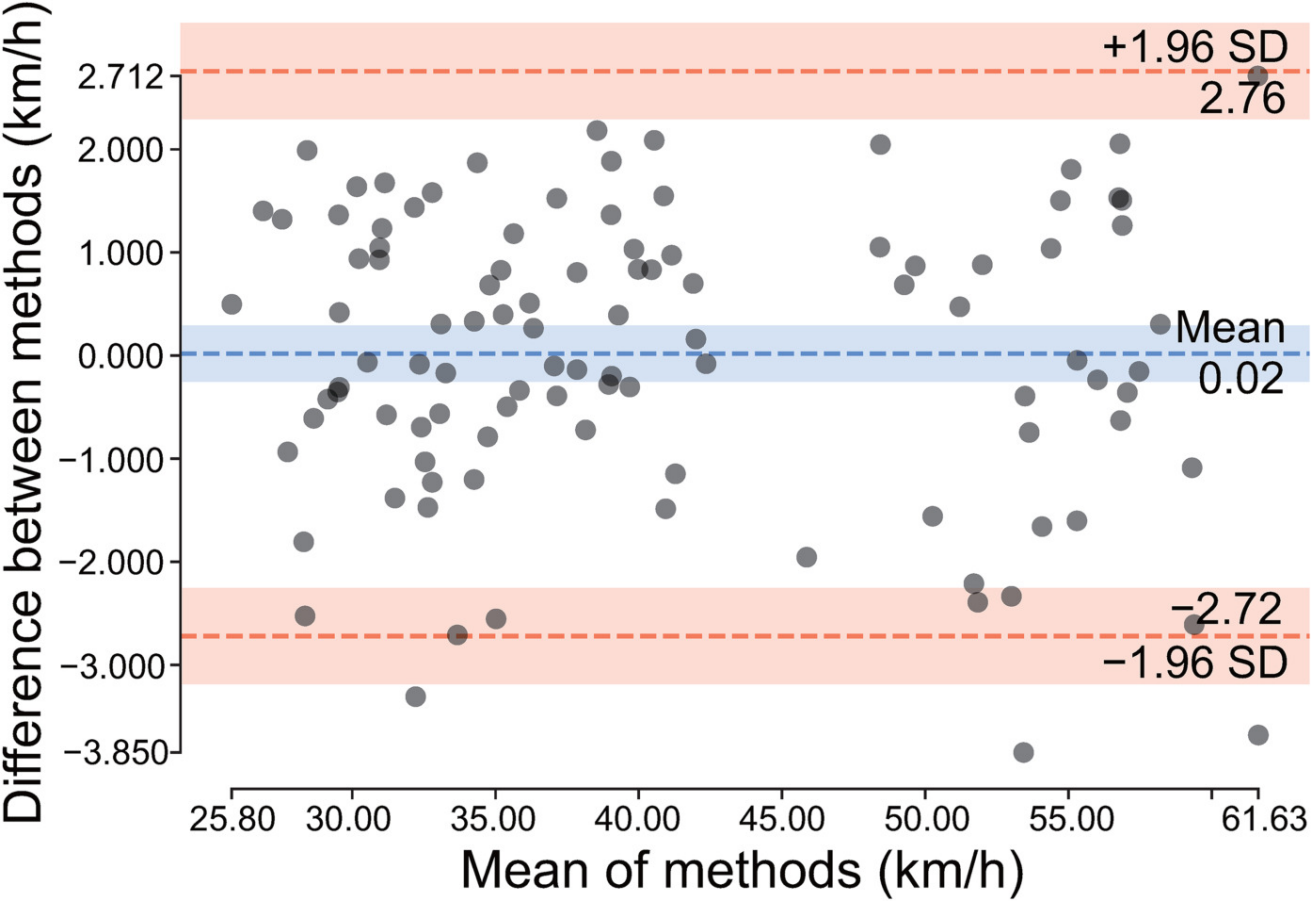
Bland–Altman analysis of predicted vs. measured ball speeds. Dotted lines indicate limits of agreement (±1.96 SD), with shaded areas representing 95% confidence intervals for the mean difference and limits of agreement.

Bland–Altman analysis demonstrated excellent agreement between predicted and measured speeds in the test set. Most data points fell within 95% confidence limits with minimal systematic bias of 0.02 km/h (Figure 2). This validated SVM model was employed for lightweight ball speed prediction.

### 2.2 Participants and protocol

This study included 34 female table tennis players (aged 9.1–21.7 years) from provincial teams training at Nanjing Sport Institute in February 2023. Twenty-six were from the Jiangsu team and eight from the Shanghai team. (Table 1). Power analysis using PASS 2023 demonstrated adequate statistical power for within-subject correlations (93%, ICC = 0.5) and between-subject correlations (87%, r = 0.5) at α= 0.05. All participants were physically fit with no training contraindications. The Ethics Committee at Nanjing Sport Institute approved these procedures (Approval No. RT-2023-02). Participants or their guardians provided written informed consent before the study began.

**Table 1:** Participant characteristics, means±SD or N (%).

Characteristics	Participants
N	34
Age (yr)	15.0±3.5
Height (cm)	161.2±9.5
Weight (kg)	51.4±10.1
Left-handed	8 (23.5%)
Right-handed	26 (76.5%)

**Figure 3:**
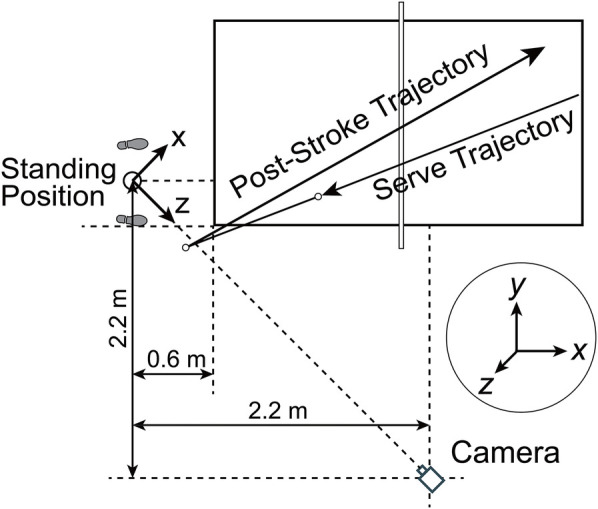
Camera positioning and coordinate system for right-handed players. The MediaPipe coordinate system was modified such that the x and z axes lie parallel to the floor plane and perpendicular to each other, while the y-axis points upward, perpendicular to the transverse plane.

An experienced coach delivered balls at slow speed (approximately 24 km/h) to allow players adequate physical and mental preparation based on her movements and optimal shot timing. Right-handed players positioned themselves on the table’s left side and executed forehand strokes toward the opposite corner, maintaining ball trajectory approximately 20 cm above the table surface. A single camera (SONY FDR-AX700) positioned 95 cm above ground level captured body movements and ball trajectories from a $45^{\circ }$ angle on the right front (Figure 3). The camera operated at 1920×1080 pixel resolution and 100 fps frame rate. The experimental setup was mirrored across the net for left-handed players: participants stood on the table’s right side, and the camera position was correspondingly adjusted. The first ten valid strokes per player–excluding edge and net contacts–were recorded, resulting in a total of 340 strokes across 34 participants. Subsequent processing removed 20 strokes due to ball speed outliers, leaving 320 strokes (8–10 per player). The outlier was identified using the criterion of values exceeding Q3+1.5×IQR or falling below Q1−1.5×IQR, where Q1 and Q3 represent first and third quartiles, respectively, and IQR denotes the interquartile range, reflecting the spread within the middle 50% of data. Finally, for correlation analysis, only the fastest and slowest two strokes per player (68 strokes in total) were selected.

### 2.3 Landmark tracking and data processing

#### 2.3.1 Human landmark tracking

**Figure 4:**
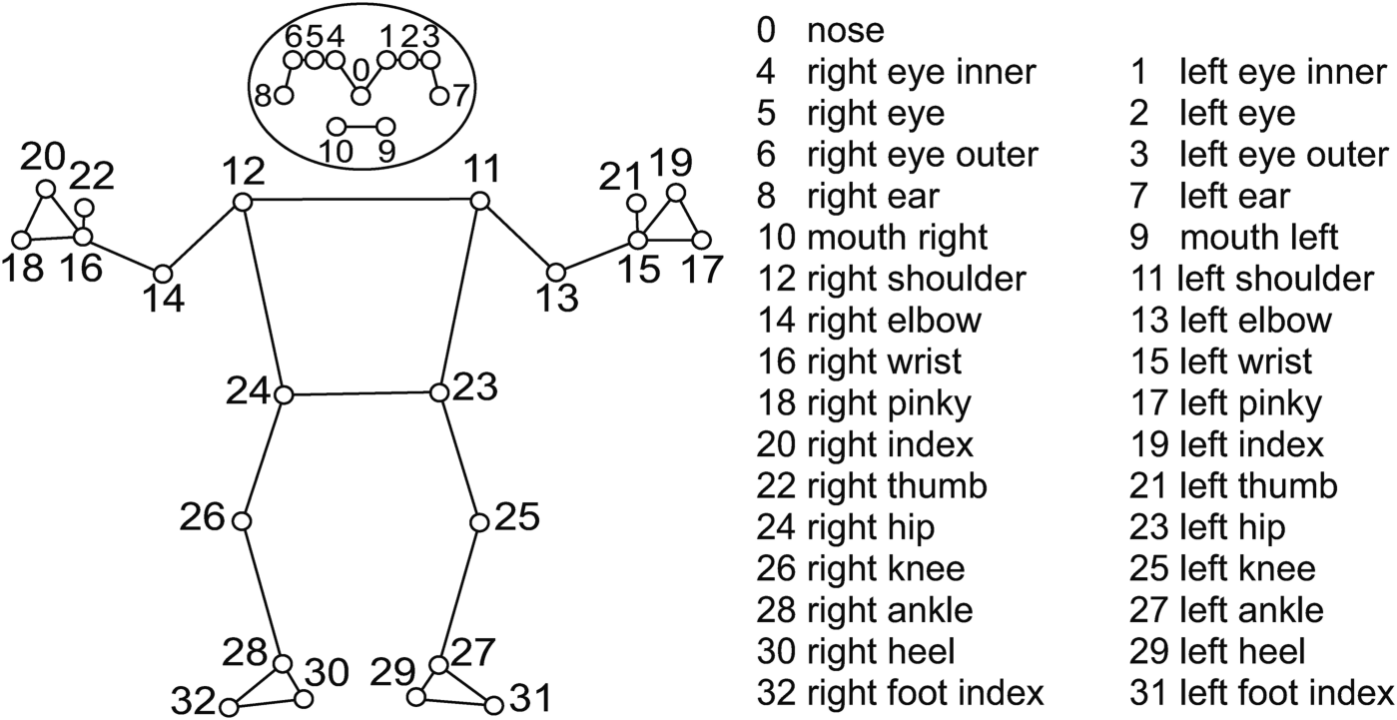
MediaPipe landmarks.

We tracked thirty-three landmark positions for each player using MediaPipe Pose (Version 0.10.14), as defined by Bazarevsky et al. (39) (Figure 4). Each landmark was represented by three-dimensional coordinates (x, y, z), where x and y indicate relative position in the 2D image, and z reflects regressed depth value. Data were stored as time series sampled at 100 Hz, matching the video frame rate. Left-handed players’ video frames were horizontally flipped before MediaPipe tracking to maintain consistency with right-handed movement patterns.

#### 2.3.2 Ball trajectory and impact timing annotation

To ensure the progression of the main experiment, this study employed manual annotation to identify ball flight trajectories and racket-ball impact timing. Ball trajectories were determined by marking the coordinates of the ball in the final six frames before it exited the video frame boundaries. These trajectory data were subsequently used to predict ball speed using the pre-trained Support Vector Machine (SVM) model. Meanwhile, the manually identified racket-ball impact timing served as temporal reference points to extrac forward swing phase.

#### 2.3.3 Position data filtering

Landmark position time series were filtered using a low-pass Finite Impulse Response (FIR) filter designed with a Hamming window and 31 taps, implemented through the scipy.signal.firwin function. The filter applied 100 Hz sampling rate and 0.08 normalized cutoff frequency, corresponding to 4 Hz actual cutoff.

#### 2.3.4 Position data scaling

Two scaling factors converted landmark coordinates from video frames to actual positions. The first factor, calculated solely for the initial frame, addresses disparity between MediaPipe-estimated body dimensions and actual measurements. It compares cumulative lengths of bilateral shoulder-hip-knee-ankle segments calculated by MediaPipe with manually measured lengths. The second factor, computed for every subsequent frame, compensates for apparent size variations due to body movement, maintaining proportional consistency across frames. This factor adjusts landmark positions by comparing cumulative segment lengths in the current frame to those in the first frame. Each landmark position is then multiplied by both factors to obtain real-world coordinates.

#### 2.3.5 Dynamic origin calibration

MediaPipe’s coordinate system is based on the camera coordinate system, which originally placed its origin (0,0,0) between the hips, with the x-axis extending rightward, y-axis downward, and z-axis toward the camera. We still use the camera coordinate system, but repositioned the origin to the time-averaged midpoint between both feet across all frames during the current forehand stroke and redefined the y-axis to extend upward (Figure 3). Landmark coordinates were then transformed relative to this motion-adaptive origin.

#### 2.3.6 Kinematic parameter calculation

**Landmark velocities** were computed from position time series using the central difference method. Velocity components in x-, y-, and z-directions were calculated from Equation 1:

(1) $$v_{x}=\frac{x_{t+1}-x_{t-1}}{2\Delta t},\;v_{y}=\frac{y_{t+1}-y_{t-1}}{2\Delta t},\;v_{z}=\frac{z_{t+1}-z_{t-1}}{2\Delta t},$$

where x, y, and z represent each landmark’s camera coordinates.

**Body segments**, defined as anatomical regions between two landmarks, were analyzed for orientation angles in the xy-, yz-, and zx-planes. Angles were computed from Equation 2:

(2) $$\alpha =\arctan\left(\frac{y}{x}\right),\;\beta =\arctan\left(\frac{z}{y}\right),\;\gamma =\arctan\left(\frac{x}{z}\right),$$

where x, y, and z denote landmark’s camera coordinates. The xy-plane is perpendicular to the camera’s optical axis, the yz-plane is parallel to the camera’s optical axis, and the zx-plane is parallel to the floor.

Angular velocities in each plane ($\omega_{xy}$, $\omega_{yz}$, $\omega_{zx}$) were derived using Equation 3:

(3) $$\omega_{xy}=\frac{\alpha_{t+1}-\alpha_{t-1}}{2\Delta t},\;\omega_{yz}=\frac{\beta_{t+1}-\beta_{t-1}}{2\Delta t},\;\omega_{zx}=\frac{\gamma_{t+1}-\gamma_{t-1}}{2\Delta t}.$$

**Joint angles** were defined as angles between two adjacent body segments, determined by three landmarks (e.g., points A, B, and C, with B at the joint). Joint angle θ was calculated from vectors $\vec{BA}$ and $\vec{BC}$ using Equation 4:

(4) $$\cos\theta =\frac{\vec{BA}\cdot \vec{BC}}{\left\|\vec{BA}\|\cdot \|\vec{BC}\right\|},$$

with corresponding angular velocity computed from Equation 5:

(5) $$\omega =\frac{\theta_{t+1}-\theta_{t-1}}{2\Delta t}.$$

#### 2.3.7 Forward swing phase extraction

**Figure 5:**
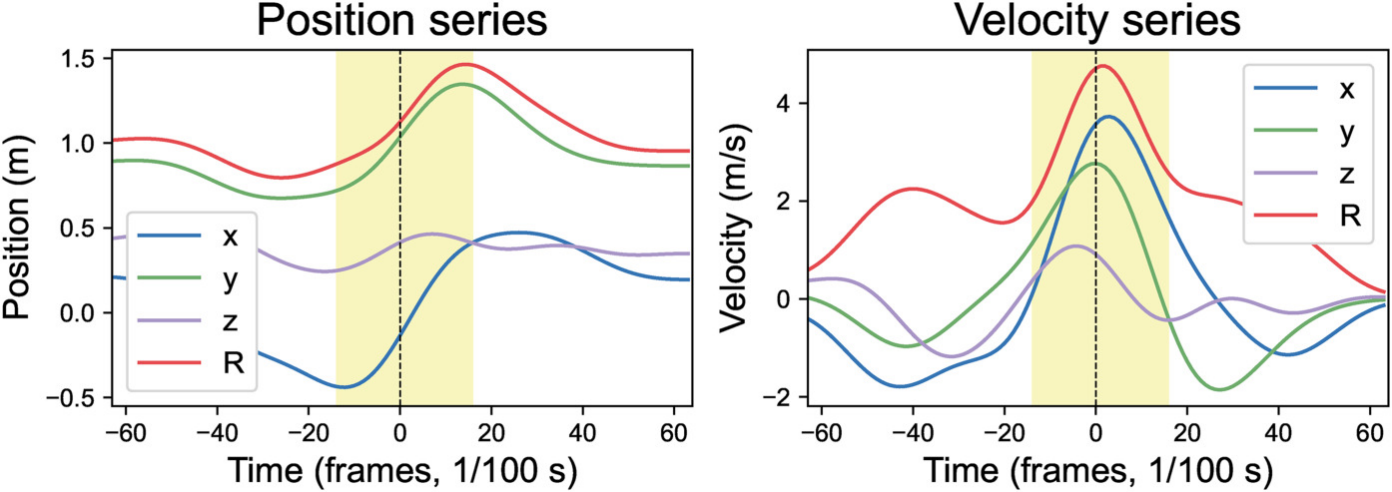
Forward swing phase in position and velocity time series. R = resultant values; x, y, z = components (x: rightward, y: upward, z: toward camera). All series are time-aligned to ball impact (vertical dashed line). Shaded area indicates the mathematically extracted forward swing phase.

A forehand stroke consists of three distinct phases: backswing, forward swing, and follow-through. This study focuses exclusively on the forward swing phase, defined as the interval during which players maximally accelerate the racket toward the ball to achieve precise contact. Forward swing duration was extracted from a duplicate playing-side wrist (Landmark 16) resultant velocity time series. The series was processed using the previously applied 31st-order FIR filter again, and its second derivative was calculated to identify inflection points. The inflection point immediately before ball impact marked phase start time, while the inflection point directly after impact defined its end time. All landmark position and velocity time-series data were then truncated based on calculated boundaries. Taking the playing-side wrist (Landmark 16) as an example, Figure 5 displays its position and velocity time series, with the shaded portion representing the truncated forward swing phase. The duration of the forward stroke phase across all players is 30.34±2.03 frames or 303.4±20.3 milliseconds.

We compared the mathematically derived start and end points of the forward swing phase with manually annotated timepoints obtained through frame-by-frame expert analysis (n = 320). The validation results demonstrate excellent agreement between the two methods. Bland–Altman analysis showed that >95% of measurements fell within the limits of agreement for both start and end time detection. Both timepoint detections exhibited excellent reliability (ICC: 0.968 and 0.987) and strong correlations with manual annotation (r: 0.974 and 0.988, both p<0.001). The mathematical method showed minimal systematic bias with mean differences of −1.016 ms for start time and −0.278 ms for end time. Mean relative errors were low at 3.69% and 1.46% respectively (Table 2).

**Table 2:** Comparison of mathematical method and manual measurement for forward swing phase timing detection.

Statistical measure	Start time	End time
Sample size (n)	320	320
Mean difference (95% CI)	−1.016 (−1.246, −0.785)	−0.278 (−0.440, −0.116)
Limits of agreement (LoA)	−5.140 to 3.109	−3.182 to 2.625
Within LoA (%)	96.2	95.3
ICC (95% CI)	0.968 (Excellent)	0.987 (Excellent)
Pearson correlation (r)	0.974 (p<0.001)	0.988 (p<0.001)
Spearman correlation (ρ)	0.958 (p<0.001)	0.981 (p<0.001)
MAE (frames, 1/100 s)	1.716	1.109
RMSE (frames, 1/100 s)	2.337	1.507
Mean relative error (%)	3.69	1.46

Note: Comparison between automated mathematical algorithm and manual expert measurement for forward swing phase timing detection. CI, confidence interval; LoA, limits of agreement (Bland–Altman analysis); ICC, intraclass correlation coefficient; MAE, mean absolute error; RMSE, root mean square error. Negative mean differences indicate earlier detection by the mathematical method. ICC interpretation: excellent (>0.90), good (0.75–0.90), moderate (0.50–0.75), poor (<0.50). All correlations are statistically significant (p<0.001).

### 2.4 Data validation

#### 2.4.1 Repeated measurement reliability

To compare the consistency and similarity of repeated position time series measurements, all position time series were truncated to a uniform duration of 0.8 s, which was slightly shorter than the minimum stroke length observed across all participants. Two strokes were randomly selected per player. Three metrics were employed for consistency and similarity validation: the intraclass correlation coefficient (ICC), normalized Dynamic Time Warping (DTW) similarity, and cosine similarity (CS). For each participant, these three similarity measures were computed for their stroke pairs, and overall similarity values were calculated as the mean across all participants.

The ICC measures reliability and consistency between repeated measurements, calculated as: $ICC=\frac{MS_{B}-MS_{W}}{MS_{B}+(k-1)MS_{W}}$ where $MS_{B}$ represents between-subjects mean square, $MS_{W}$ represents within-subjects mean square, and k represents the number of measurements per subject.

Normalized DTW similarity captures temporal alignment between sequences with potential time warping, computed as: $S_{DTW}=\frac{1}{1+\frac{DTW(X,Y)}{L_{avg}\cdot R_{data}}}$ where $L_{avg}$ represents average sequence length and $R_{data}$ denotes data range.

Cosine similarity measures angular similarity between vectors, defined as: $S_{cos}=1-\frac{X\cdot Y}{\left\|X\|\cdot \|Y\right\|}$.

Table 3 demonstrate strong measurement consistency across different similarity metrics. Most landmarks achieved excellent reliability (⩾0.90) in multiple dimensions, confirming the robustness of the motion capture model and the consistency of skilled athletic performance.

**Table 3:** Consistency and similarity measures for repeated landmark position measurements: intraclass correlation coefficients (ICC), normalized DTW similarity, and cosine similarity.

LM	ICC				Normalized DTW similarity				Cosine similarity			
	R	x	y	z	R	x	y	z	R	x	y	z
0	0.982^a^	0.909^a^	0.978^a^	0.860	0.900	0.985^a^	0.953^a^	0.987^a^	1.000^a^	0.815	1.000^a^	0.985^a^
11	0.983^a^	0.907^a^	0.982^a^	0.849	0.941^a^	0.967^a^	0.958^a^	0.991^a^	1.000^a^	0.895	1.000^a^	0.936^a^
12	0.982^a^	0.843	0.978^a^	0.843	0.961^a^	0.988^a^	0.967^a^	0.976^a^	1.000^a^	0.874	1.000^a^	0.963^a^
13	0.895	0.886	0.939^a^	0.831	0.981^a^	0.976^a^	0.984^a^	0.992^a^	0.998^a^	0.897	0.998^a^	0.907^a^
14	0.952^a^	0.836	0.945^a^	0.866	0.989^a^	0.993^a^	0.989^a^	0.982^a^	0.999^a^	0.944^a^	0.998^a^	0.957^a^
15	0.901^a^	0.901^a^	0.918^a^	0.854	0.983^a^	0.982^a^	0.985^a^	0.991^a^	0.991^a^	0.841	0.992^a^	0.863
16	0.930^a^	0.798	0.911^a^	0.857	0.993^a^	0.995^a^	0.993^a^	0.975^a^	0.997^a^	0.963^a^	0.996^a^	0.935^a^
23	0.982^a^	0.803	0.986^a^	0.910^a^	0.782	0.976^a^	0.775	0.973^a^	1.000^a^	0.932^a^	1.000^a^	0.847
24	0.987^a^	0.712	0.984^a^	0.926^a^	0.821	0.977^a^	0.772	0.976^a^	1.000^a^	0.976^a^	1.000^a^	0.990^a^
25	0.915^a^	0.905^a^	0.893	0.817	0.976^a^	0.956^a^	0.983^a^	0.983^a^	0.996^a^	0.991^a^	0.996^a^	0.764
26	0.885	0.862	0.916^a^	0.847	0.980^a^	0.983^a^	0.975^a^	0.971^a^	0.998^a^	0.904^a^	0.999^a^	0.991^a^
27	0.796	0.863	0.738	0.763	0.966^a^	0.954^a^	0.980^a^	0.970^a^	0.992^a^	0.991^a^	0.889	0.978^a^
28	0.835	0.858	0.731	0.824	0.968^a^	0.954^a^	0.978^a^	0.969^a^	0.994^a^	0.993^a^	0.851	0.966^a^

Note: LM, landmark number; x, y, z, displacement components in camera coordinate system; $R=\sqrt{x^{2}+y^{2}+z^{2}}$; ICC, intraclass correlation coefficient; DTW, dynamic time warping. ^a^excellent⩾0.90.

**Figure 6:**
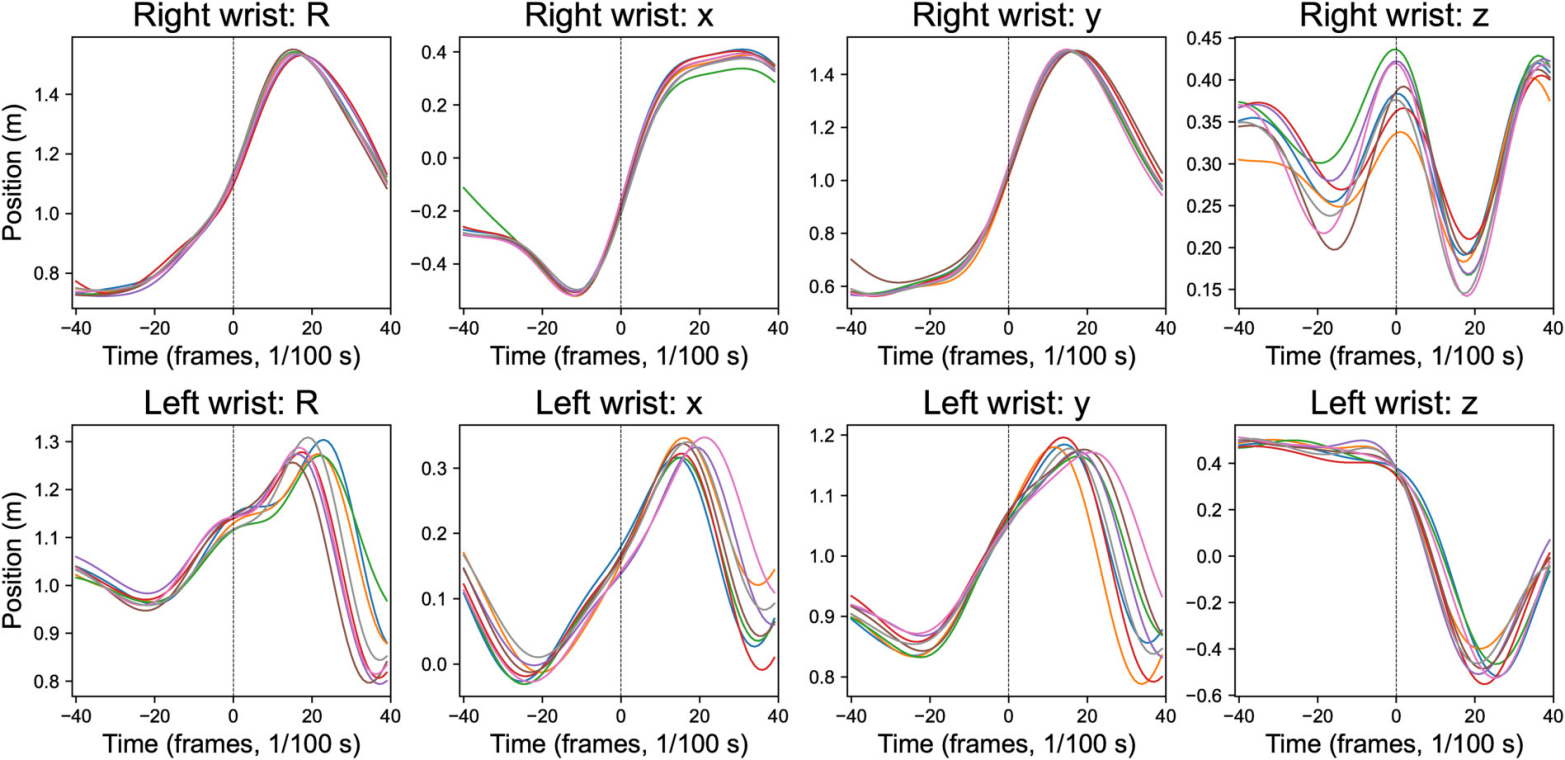
Wrist position time series for a single participant across multiple trials. x, y, z = position components (x: rightward, y: upward, z: toward the camera), and $R=\sqrt{x^{2}+y^{2}+z^{2}}$. Lines of different colors represent the eight trials. All time series are aligned to ball impact (vertical dashed line).

Figure 6 demonstrates a single player’s position trajectories across multiple trials, revealing high consistency, particularly in the dominant-side wrist’s x and y coordinates, while the z-coordinate shows greater variability. This consistent performance across trials reflects both the movement reliability of professionally trained players and MediaPipe’s consistency and similarity in repeated assessments.

#### 2.4.2 Left-handed data validation

Left-handed video frames were horizontally flipped before MediaPipe processing to generate coordinate data comparable to right-handed players. Landmark position curve analysis employed multiple statistical approaches to assess group differences. Curves were preprocessed through cubic spline interpolation to achieve uniform temporal resolution. Eighteen kinematic features were extracted, including basic statistics (mean, standard deviation, maximum, minimum, range, median), distribution characteristics (skewness, kurtosis), peak properties (peak count, peak maximum, peak mean), temporal dynamics (mean velocity, maximum acceleration, zero crossings), spectral properties (dominant frequency, spectral centroid), and curve morphology (curve length, area under curve). Statistical comparisons utilized appropriate parametric or non-parametric tests based on normality and variance assessments. Bonferroni correction addressed multiple comparison issues. Multivariate analysis included dimensionality reduction and clustering for pattern recognition. Curve similarity was quantified using distance matrices comparing intra-group vs. inter-group variations.

**Figure 7:**
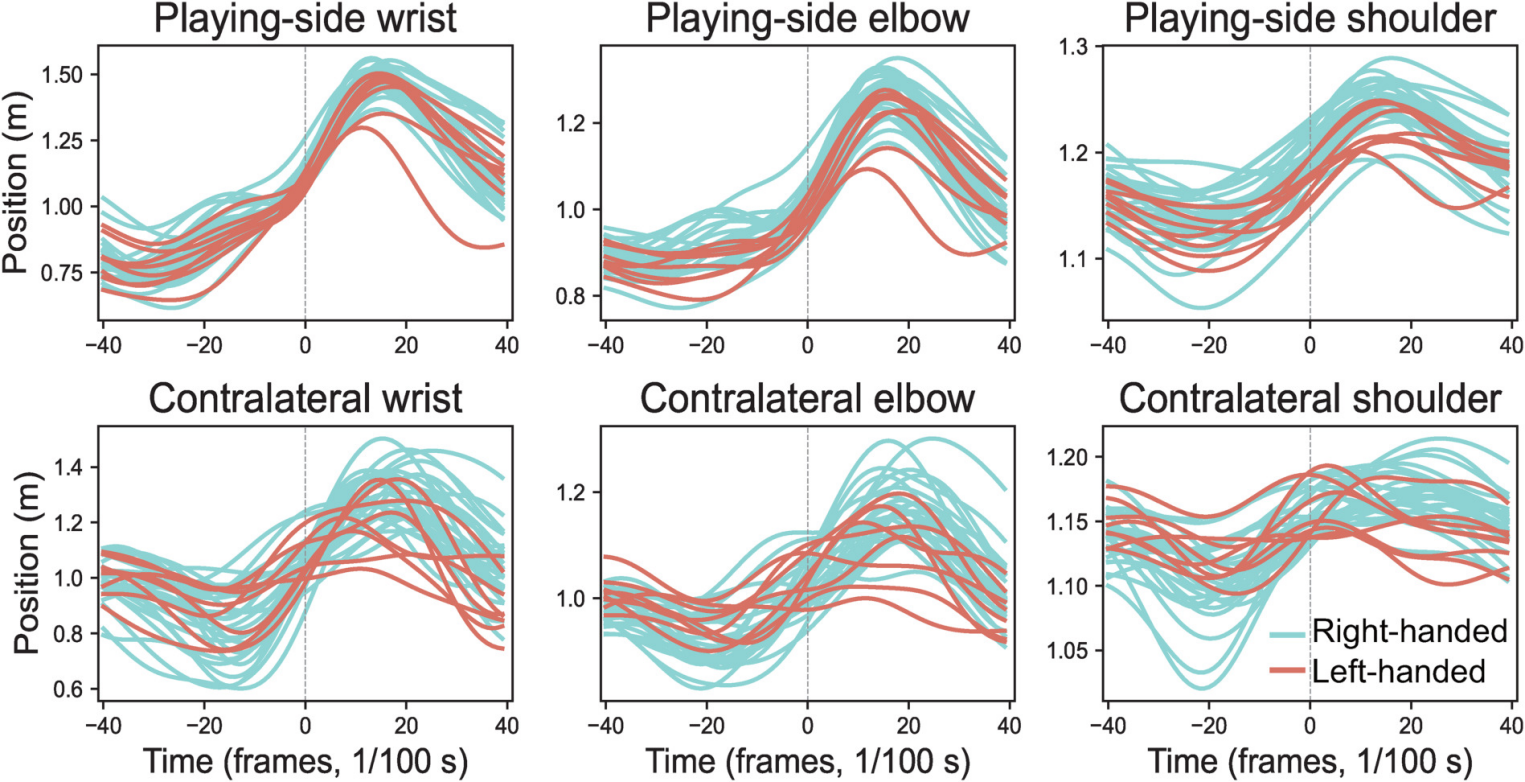
Average resultant position curves for left-handed (n = 8, coral pink) vs. right-handed (n = 26, turquoise) players, aligned to ball impact (dashed line).

Results demonstrated no significant differences in resultant position curves for all 33 landmarks between left-handed and right-handed players (corrected p>0.05), suggesting both groups originated from the same population. This finding validates the comparability of left-handed and right-handed player data. Figure 7 illustrates the consistent resultant position curves between groups for playing-side wrist, elbow, shoulder, and contralateral wrist, elbow, shoulder landmarks.

### 2.5 Statistical analysis

The fastest and slowest strokes from each player were paired and analyzed using two correlation approaches: within-subject and between-subject. We chose correlation analysis over machine learning approaches for several reasons: (1) it provides direct, interpretable relationships between biomechanical variables that coaches and researchers can immediately understand and apply; (2) correlation analysis is statistically appropriate and robust for small sample sizes; and (3) this exploratory approach helps identify which biomechanical factors are most relevant before developing more complex predictive models.

#### 2.5.1 Within-subject corrilation

Within-subject correlation is a statistical method used to assess association between paired measures across multiple occasions for each individual (40). This technique examines whether an increase in one variable corresponds to change in another (41). For example, it determines whether faster wrist movement correlates with higher ball speeds.

Statistical summaries of positional and angular changes are provided in Table 4. These kinematic parameters were extracted across three levels: (1) Landmark parameters including positional range (PR), mean velocity (MV), peak velocity (PV), and impact velocity (IV), decomposed into resultant components (subscript R) and axial components (subscripts x, y, z); (2) Segment parameters including angular range (AR), mean angular velocity (MAV), peak angular velocity (PAV), and impact angular velocity (IAV), analyzed as resultant components (subscript R) and planar components (subscripts xy, yz, zx); (3) Joint parameters including joint angular range (JAR), joint mean angular velocity (JMAV), joint peak angular velocity (JPAV), and joint impact angular velocity (JIAV), treated as scalar quantities without directional decomposition. Within-subject correlations (*r*_ws_) between kinematic parameters and ball speeds were calculated using repeated measures correlation analysis (Pingouin.rm_corr function), following the rmcorr methodology of Bakdash and Marusich (42) (Equation 6).

(6) $$r_{\text{rm}}=\sqrt{\frac{SS_{\text{Measure}}}{SS_{\text{Measure}}+SS_{\text{Error}}}}$$

$$\text{Sign of }r_{\text{rm}}\text{ ( positive or negative) }=\text{Sign of }\beta$$

where $SS_{\text{Measure}}$ represents the sum of squares for the measure (dependent variable), $SS_{\text{Error}}$ represents the sum of squares for error (residual variance), and β represents the common regression slope coefficient estimated across all participants, estimated through analysis of covariance (ANCOVA).

**Table 4:** Summary of kinematic variables during the forward swing phase.

Summaries	Descriptions
Positional/angular range	Total absolute positional or angular change during forward swing
Mean velocity	Absolute mean linear or angular velocity during forward swing
Peak velocity	Absolute peak linear or angular velocity during forward swing
Impact velocity	Absolute linear or angular velocity at racket-ball impact

For each player, the fastest stroke was compared with the slowest stroke to compute *r*_ws_. This method aimed to identify key factors driving differences between fastest and slowest ball speeds.

#### 2.5.2 Between-subject corrilation

Between-subject correlation evaluates whether individuals with higher values in one variable tend to exhibit higher values in another across subjects (41). For example, it can assess whether age or height correlates with ball speed. The between-subject correlation coefficient (*r*_bs_) is calculated using Equation 7 proposed by Bland and Altman (41):

(7) $$r=\frac{\sum m_{i}\bar{x_{i}}\bar{y_{i}}-\frac{\sum m_{i}\bar{x_{i}}\cdot \sum m_{i}\bar{y_{i}}}{\sum m_{i}}}{\sqrt{\left(\sum m_{i}{\bar{x_{i}}}^{2}-\frac{{\left(\sum m_{i}\bar{x_{i}}\right)}^{2}}{\sum m_{i}}\right)\left(\sum m_{i}{\bar{y_{i}}}^{2}-\frac{{\left(\sum m_{i}\bar{y_{i}}\right)}^{2}}{\sum m_{i}}\right)}}$$

where $m_{i}$ is the number of measurements for subject i (stroke count for a given player), and $\bar{x_{i}}$ and $\bar{y_{i}}$ are the means of two variables across multiple measurements for subject i.

## 3 Results

### 3.1 Within-subject correlation between biomechanical kinematic characteristics and ball speed

#### 3.1.1 Relationship between positional range, linear velocity of landmarks, and ball speed

**Figure 8:**
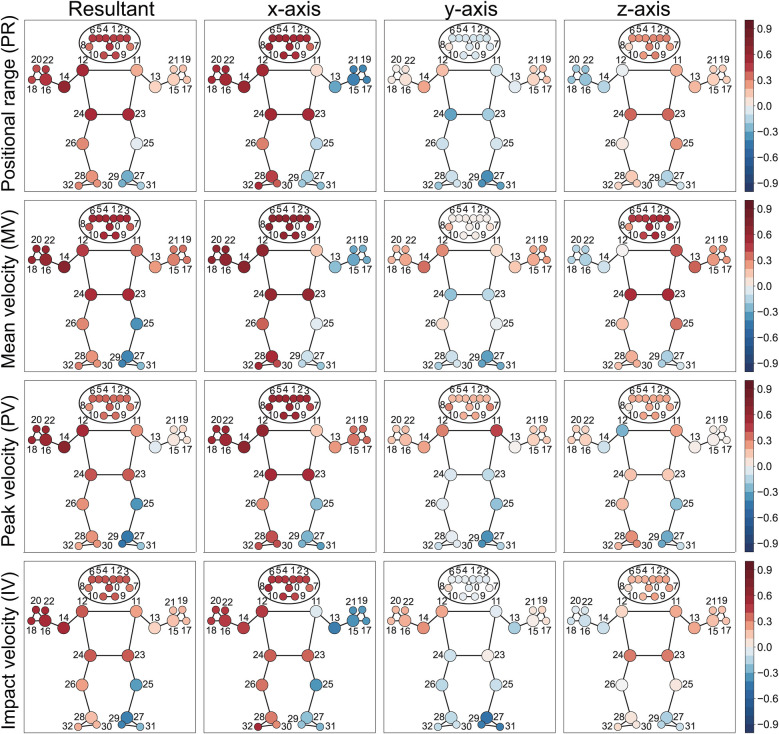
Heatmap visualization of within-subject correlation between landmark kinematics and ball speed in the camera coordinate system, where the x-axis points right, y-axis points up and z-axis points toward the camera (Figure 3). The resultant value represents the square root of the sum of squared components from the x, y, and z axes. Colored circles indicate significant correlations, with red representing positive correlations and blue representing negative correlations. Numbers indicate anatomical landmarks.

The positional range and velocities (mean, peak, and at impact) of the head, playing-side shoulder (*r*_ws_ = 0.51, 0.63, 0.61 for PR_x_, MV_x_, PV_x_), elbow (*r*_ws_ = 0.63, 0.70, 0.69 for PR_R_, MV_R_, PV_R_), wrist (*r*_ws_ = 0.60, 0.56, 0.50 for MV_R_, PV_R_, IV_R_), fingers, and both hips show significant positive correlations with ball speed, particularly along the x-axis. The resultant peak and impact velocities of the legs reveal distinct patterns: the playing-side knee shows a weak positive correlation, while the contralateral knee and ankle display weak to moderate negative correlations with ball speed (Figure 8 and Table 5).

**Table 5:** Within-subject correlation coefficients (*r*_ws_) between landmark kinematic variables (positional range and velocities) and ball speed.

LM	Positional range (PR)				Mean velocity (MV)				Peak velocity (PV)				Impact velocity (IV)			
	R	x	y	z	R	x	y	z	R	x	y	z	R	x	y	z
0	0.47^b^	0.52^b^	−0.06	0.30	0.53^b^	0.62^b^	0.02	0.48^b^	0.38^a^	0.56^b^	0.20	0.22	0.42^a^	0.46^b^	−0.04	0.22
11	0.21	0.08	−0.07	0.21	0.35^a^	0.15	0.09	0.40^a^	0.27	0.14	0.44^b^	0.24	0.24	−0.05	−0.04	0.22
12	0.50^b^	0.51^b^	0.17	−0.03	0.45^b^	0.63^b^	0.28	0.01	0.51^b^	0.61^b^	0.30	−0.27	0.39^a^	0.46^b^	0.22	0.11
13	0.12	−0.30	−0.04	0.18	0.25	−0.23	0.14	0.38^a^	−0.05	0.26	0.01	0.03	0.11	−0.43^a^	−0.17	0.22
14	0.63^b^	0.59^b^	0.23	−0.15	0.70^b^	0.68^b^	0.35^a^	−0.10	0.69^b^	0.65^b^	0.23	−0.10	0.50^b^	0.44^b^	0.27	−0.10
15	0.12	−0.41^a^	0.11	0.12	0.30	−0.30	0.21	0.25	0.05	0.33	0.12	0.02	0.16	−0.36^a^	0.02	0.16
16	0.46^b^	0.52^b^	0.06	−0.21	0.60^b^	0.64^b^	0.22	−0.16	0.56^b^	0.55^b^	0.17	0.12	0.50^b^	0.46^b^	0.20	−0.10
17	0.15	−0.40^a^	0.15	0.13	0.32	−0.29	0.24	0.25	0.07	0.36^a^	0.14	0.02	0.18	−0.36^a^	0.07	0.16
18	0.43^a^	0.49^b^	0.04	−0.21	0.56^b^	0.61^b^	0.19	−0.15	0.53^b^	0.52^b^	0.15	0.13	0.49^b^	0.45^b^	0.17	−0.09
19	0.16	−0.40^a^	0.17	0.13	0.34^a^	−0.28	0.25	0.25	0.10	0.36^a^	0.16	0.04	0.19	−0.35^a^	0.10	0.16
20	0.41^a^	0.48^b^	0.02	−0.19	0.57^b^	0.62^b^	0.17	−0.15	0.53^b^	0.52^b^	0.13	0.13	0.50^b^	0.46^b^	0.18	−0.04
21	0.13	−0.41^a^	0.13	0.13	0.32	−0.30	0.22	0.25	0.07	0.33	0.13	0.03	0.17	−0.36^a^	0.05	0.16
22	0.45^b^	0.51^b^	0.05	−0.21	0.59^b^	0.63^b^	0.21	−0.16	0.55^b^	0.54^b^	0.16	0.11	0.50^b^	0.46^b^	0.20	−0.07
23	0.49^b^	0.51^b^	−0.19	0.36^a^	0.49^b^	0.59^b^	−0.14	0.49^b^	0.38^a^	0.50^b^	−0.13	0.14	0.38^a^	0.37^a^	0.03	0.32
24	0.49^b^	0.51^b^	−0.32	0.37^a^	0.48^b^	0.60^b^	−0.22	0.48^b^	0.40^a^	0.50^b^	−0.06	0.16	0.38^a^	0.38^a^	−0.10	0.32
25	−0.04	−0.14	−0.08	0.27	−0.36^a^	−0.04	−0.02	0.34^a^	−0.36^a^	−0.24	−0.22	−0.23	−0.33	−0.35^a^	−0.11	0.05
26	0.28	0.31	−0.10	0.04	0.29	0.38^a^	0.08	0.16	0.27	0.27	−0.04	0.17	0.24	0.33	−0.14	0.00
27	−0.28	−0.16	−0.37^a^	−0.16	−0.40^a^	−0.09	−0.32	−0.17	−0.45^b^	−0.26	−0.37^a^	−0.30	−0.42^a^	−0.23	−0.47^b^	−0.17
28	0.26	0.44^b^	−0.11	0.14	0.26	0.49^b^	−0.11	0.20	0.27	0.41^a^	−0.03	0.28	0.17	0.31	−0.13	0.05
29	−0.28	−0.20	−0.37^a^	−0.15	−0.41^a^	−0.14	−0.31	−0.17	−0.45^b^	−0.27	−0.37^a^	−0.30	−0.41^a^	−0.21	−0.43^a^	−0.14
30	0.27	0.38^a^	−0.08	0.13	0.27	0.42^a^	−0.07	0.20	0.26	0.42^a^	−0.04	0.28	0.18	0.28	−0.10	0.05
31	−0.17	−0.23	−0.33	−0.09	−0.24	−0.20	−0.30	−0.08	−0.21	−0.33^a^	−0.15	−0.11	−0.25	−0.26	−0.43^b^	−0.11
32	0.30	0.49^b^	−0.21	0.11	0.31	0.52^b^	−0.18	0.16	0.21	0.41^a^	−0.11	0.19	0.21	0.50^b^	−0.13	0.09

x, y, z are displacement components in the camera coordinate system: x-axis points right, y-axis points up, and z-axis points toward the camera (Figure 3), $R=\sqrt{x^{2}+y^{2}+z^{2}}$. “LM” refers to Landmark (Figure 4). ^a^p<0.05. ^b^p<0.01.

#### 3.1.2 Relationship between angular range, angular velocity of body segments, and ball speed

**Figure 9:**
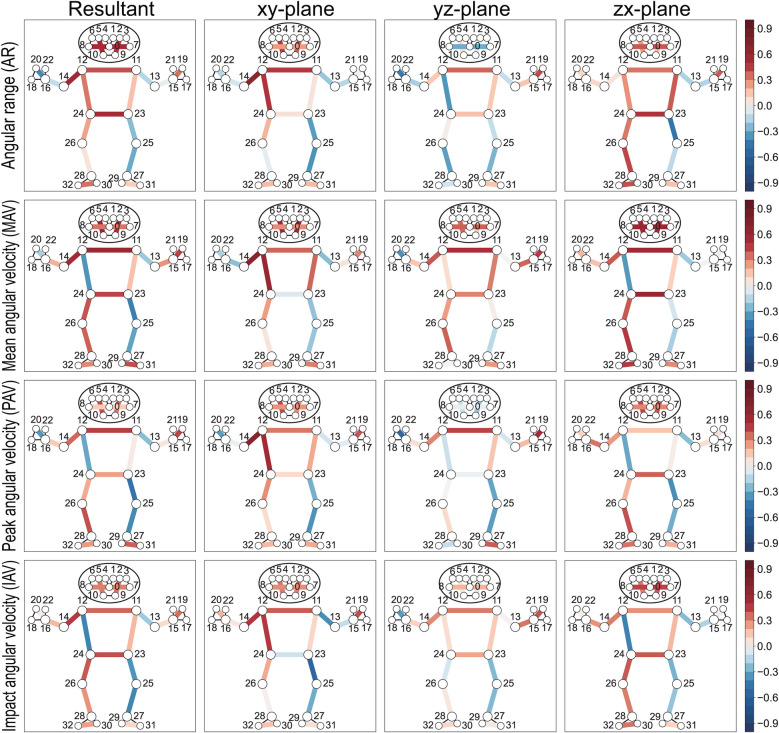
Heatmap visualization of within-subject correlation between segment kinematics and ball speed. The human skeleton model displays correlations for different kinematic components across three planar projections (xy, yz, and zx-plane) in the camera coordinate system, where the x-axis points right, y-axis points up, and z-axis points toward the camera (Figure 3). The resultant value represents the combined magnitude of these components. Colored lines indicate significant correlations, with red representing positive correlations and blue representing negative correlations. Numbers indicate anatomical landmarks.

The resultant angular range and mean angular velocity of the shoulder line (11–12) (*r*_ws_ = 0.57, 0.54 for MAV_R_, MAV_zx_) and hip line (23–24) (*r*_ws_ = 0.51, 0.59 for AR_R_, MAV_zx_) show moderate correlation with ball speed. The playing-side upper arm (12–14) demonstrates significant positive correlations in both angular range and angular velocities with ball speed (*r*_ws_ = 0.65, 0.71, 0.70 for AR_xy_, MAV_xy_, PAV_xy_), while the contralateral upper arm (11–13) shows no significant relationship. In the lower limbs, the playing-side segments (24–26, 26–28) present weak to moderately positive correlations, whereas the contralateral segments (23–25, 25–27) display weak to moderate negative correlations (Figure 9, Table 6).

**Table 6:** Within-subject correlation coefficients (*r*_ws_) between segment kinematic variables (angular range and velocities) and ball speed.

Segment	Angular range (AR)				Mean angular velocity (MAV)				Peak angular velocity (PAV)				Impact angular velocity (IAV)			
	R	xy	yz	zx	R	xy	yz	zx	R	xy	yz	zx	R	xy	yz	zx
2–9	0.46^b^	0.38^a^	−0.06	0.16	0.48^b^	0.52^b^	0.13	0.53^b^	0.32	0.27	−0.22	0.40^a^	0.34^a^	0.30	−0.06	0.40^a^
5–10	0.52^b^	0.46^b^	0.08	0.25	0.60^b^	0.54^b^	0.35^a^	0.56^b^	0.41^a^	0.42^a^	−0.12	0.43^a^	0.32	0.32	0.05	0.32
7–8	0.49^b^	0.25	−0.29	0.39^a^	0.33	0.28	0.37^a^	0.58^b^	0.13	0.26	−0.03	0.29	0.31	0.28	0.19	0.44^b^
11–12	0.41^a^	0.46^b^	0.36^a^	0.33	0.57^b^	0.36^a^	0.48^b^	0.54^b^	0.45^b^	0.32	0.44^b^	0.15	0.37^a^	0.38^a^	0.32	0.27
23–24	0.51^b^	0.06	0.16	0.47^b^	0.43^b^	−0.05	0.30	0.59^b^	0.24	0.09	−0.02	0.37^a^	0.41^a^	−0.09	0.23	0.39^a^
11–13	−0.15	−0.20	−0.12	−0.16	−0.20	−0.24	0.00	−0.19	−0.24	−0.08	−0.07	−0.24	−0.19	−0.37^a^	0.01	−0.25
12–14	0.55^b^	0.65^b^	0.14	0.05	0.52^b^	0.71^b^	0.39^a^	0.38^a^	0.36^a^	0.70^b^	0.29	0.25	0.46^b^	0.46^b^	0.28	0.31
13–15	−0.03	−0.14	0.15	−0.16	0.27	0.05	0.36^a^	−0.01	0.04	−0.04	0.17	0.07	0.09	−0.13	0.33	−0.17
14–16	−0.13	−0.08	−0.18	0.10	0.20	−0.23	0.15	0.16	0.14	−0.02	0.08	0.33	0.18	0.04	0.12	0.27
15–19	0.34^a^	0.01	0.42^a^	0.38^a^	0.44^b^	0.30	0.45^b^	−0.02	0.43^b^	0.40^a^	0.50^b^	0.07	0.35^a^	0.42^a^	0.40^a^	0.02
16–20	−0.38^a^	−0.15	−0.44^b^	0.10	−0.18	−0.17	−0.36^a^	0.19	−0.34^a^	−0.34^a^	−0.52^b^	0.12	0.03	0.19	−0.34^a^	0.02
11–23	0.16	0.05	0.15	0.38^a^	0.16	0.37^a^	0.31	0.13	0.03	0.22	0.15	0.03	0.18	0.10	0.14	0.18
12–24	0.33	0.47^b^	−0.35^a^	0.27	−0.35^a^	0.59^b^	0.08	−0.34^a^	−0.30	0.52^b^	−0.11	−0.30	−0.41^a^	0.46^b^	0.08	−0.41^a^
23–25	−0.24	−0.33	−0.12	−0.45^b^	−0.42^a^	−0.22	0.03	−0.07	−0.46^b^	−0.27	−0.31	−0.37^a^	−0.35^a^	−0.54^b^	−0.25	−0.27
24–26	0.23	0.19	0.03	0.32	0.40^a^	0.25	0.25	0.39^a^	0.21	0.27	0.00	0.19	0.40^a^	0.21	−0.07	0.39^a^
25–27	−0.29	−0.36^a^	−0.26	−0.13	−0.32	−0.21	−0.14	−0.18	−0.38^a^	−0.36^a^	−0.33	−0.28	−0.40^a^	−0.29	−0.25	−0.21
26–28	0.05	−0.03	−0.33	0.43^a^	0.37^a^	0.05	0.38^a^	0.46^b^	0.41^a^	0.08	0.09	0.41^a^	0.27	0.02	0.05	0.31
29–31	0.13	0.17	0.15	0.16	0.41^a^	0.30	0.26	0.28	0.38^a^	0.22	0.38^a^	0.20	0.11	0.07	0.04	0.12
30–32	0.34^a^	0.18	−0.09	0.41^a^	0.29	0.21	0.00	0.46^b^	0.25	0.18	−0.12	0.34^a^	0.24	0.15	0.10	0.33

Segment denotes the anatomical connection between two MediaPipe landmarks (Figure 4). R represents the resultant angular value (position or velocity), with xy, yz, and zx as planar components relative to the camera coordinate system (Figure 3): the xy-plane (perpendicular to the optical axis), yz-plane (parallel to the optical axis), and zx-plane (parallel to the floor). ^a^p<0.05. ^b^p<0.01.

#### 3.1.3 Relationship between angular range, angular velocity of joint, and ball speed

**Figure 10:**
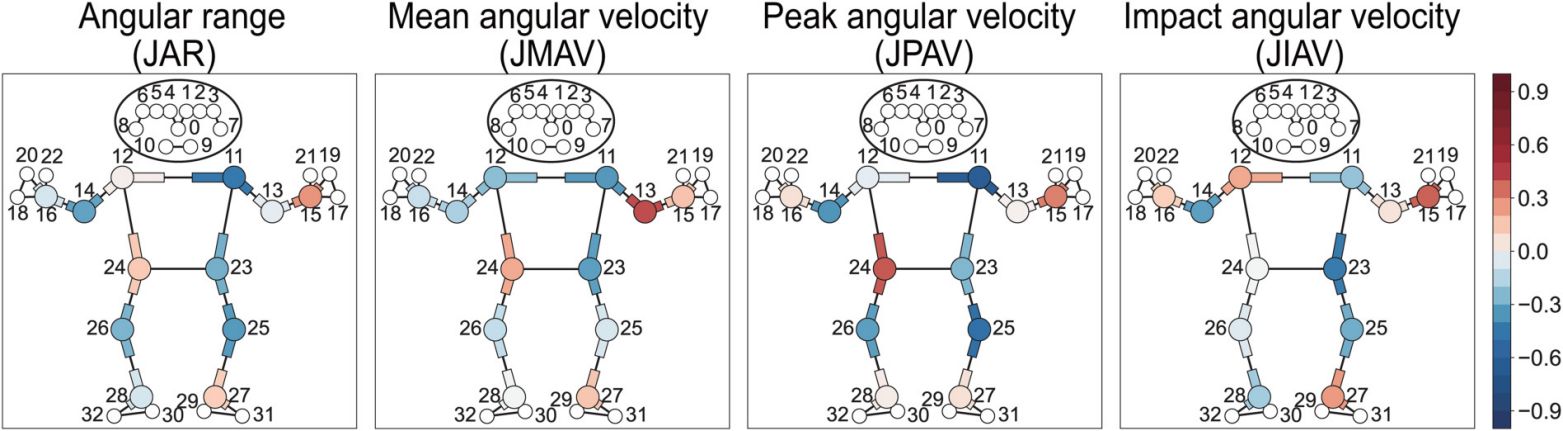
Heatmap visualization of within-subject correlation between joint kinematics and ball speed. The “J” prefix in parameter abbreviations denotes joint-related measurements. Each joint is defined by three landmarks, with the middle landmark representing the joint position. Joints and their adjacent segments are color-coded, with red representing positive correlations and blue representing negative correlations. Numbers indicate anatomical landmarks.

Negative correlations with ball speed are observed for the contralateral shoulder’s horizontal flexion-extension (*r*_ws_ = −0.44, −0.62 for joint JAR and JPAV), the playing-side elbow’s flexion-extension (*r*_ws_ = −0.35 for joint JPAV), and both knees’ flexion-extension (Figure 10, Table 7).

**Table 7:** Within-subject correlation coefficients (*r*_ws_) between joint kinematic variables (angular range and velocities) and ball speed.

Joint angle	Angular range(JAR)	Mean angular velocity (JMAV)	Peak angular velocity (JPAV)	Impact angular velocity (JIAV)
11-12-14	0.02	−0.23	−0.03	0.22
12-11-13	−0.44^b^	−0.35^a^	−0.62^b^	−0.21
12-14-16	−0.32	−0.17	−0.35^a^	−0.33
11-13-15	−0.03	0.42^a^	0.01	0.05
14-16-20	−0.07	−0.11	0.06	0.12
13-15-19	0.26	0.16	0.31	0.37^a^
12-24-26	0.14	0.21	0.39^a^	−0.00
11-23-25	−0.28	−0.32	−0.26	−0.44^b^
24-26-28	−0.26	−0.13	−0.32	−0.05
23-25-27	−0.34^a^	−0.07	−0.47^b^	−0.28
25-27-31	0.13	0.15	0.06	0.25
26-28-32	−0.07	−0.00	0.03	−0.18

Joint angles are calculated using three consecutive landmarks (Figure 4), with the middle keypoint defining the joint center. ^a^p<0.05. ^b^p<0.01.

### 3.2 Between-subject correlation coefficients of age and height with ball speed

**Figure 11:**
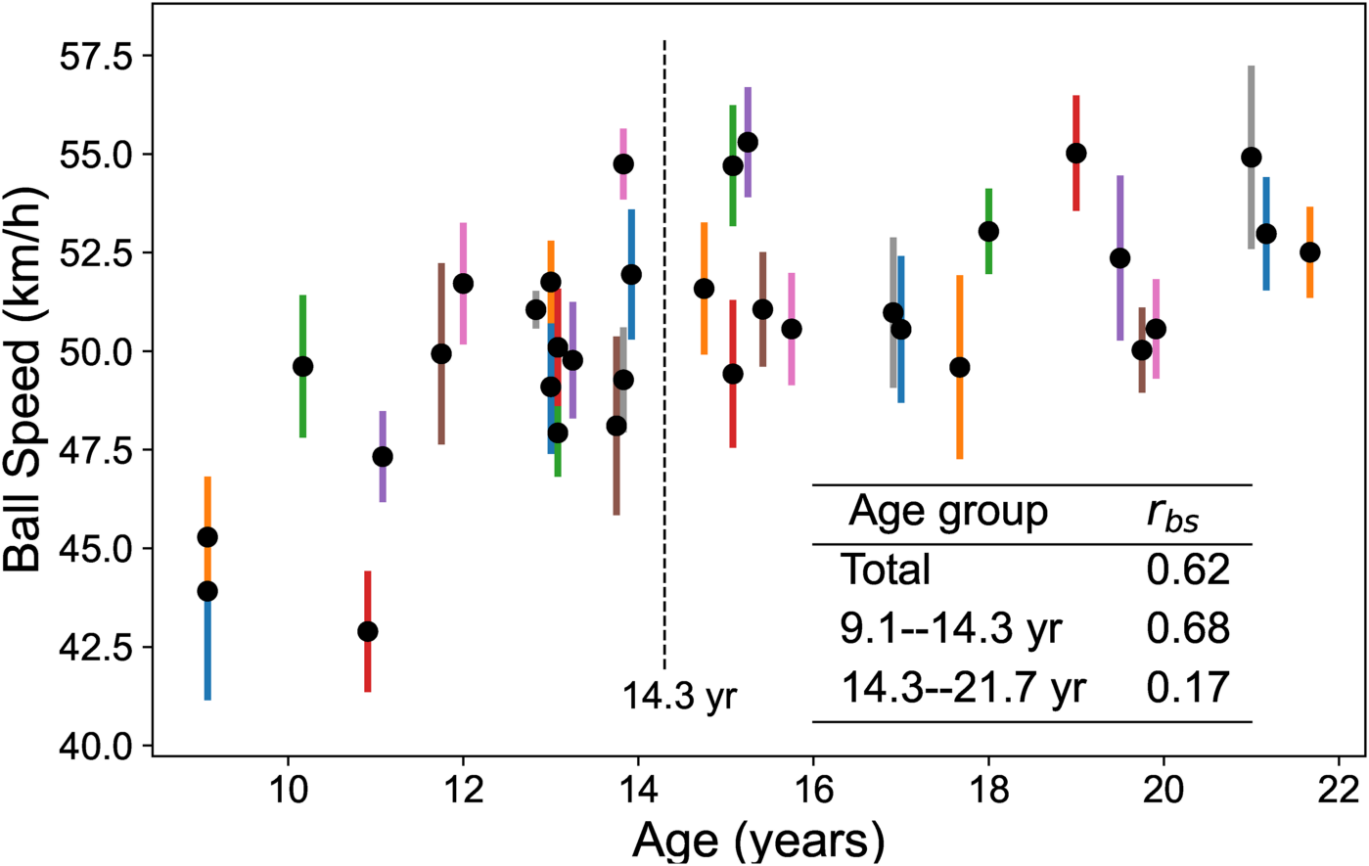
Ball speed distribution by age group. Black dots represent individual player means, colored lines show 95% confidence intervals, and *r*_bs_ denotes the between-subject correlation coefficient.

Ball speed correlates significantly with age (*r*_bs_ = 0.62), particularly in younger participants (9.1–14.3 years, *r*_bs_ = 0.68). Height also shows positive correlations with ball speed. However, among female adolescents (14.3–21.7 years), age shows weak correlation (*r*_bs_ = 0.17) while height shows slight negative correlation (*r*_bs_ = −0.29) with ball speed (Table 8, Figure 11).

**Table 8:** Between-subject correlation matrix showing relationships among age, height, and ball speed across age groups.

Participant characteristics	Total (9.1–21.7 yr)			<14.3yr			>14.3yr		
	Age	Height	BS	Age	Height	BS	Age	Height	BS
Age	–	0.61^a^	0.62^a^	–	0.87^a^	0.68^a^	–	−0.14	0.17
Height	0.61^a^	–	0.55^a^	0.87^a^	–	0.63^a^	−0.14	–	−0.29
BS	0.62^a^	0.55^a^	–	0.68^a^	0.63^a^	–	0.17	−0.29	–

BS, ball speed.

^a^p<0.01.

## 4 Discussion

### 4.1 MediaPipe-based motion capture reveals critical kinematic parameters

Our MediaPipe-based analysis revealed specific kinematic metrics that correlate with ball speed in table tennis forehand strokes. Ball speed increased with greater playing-side arm linear movement at the shoulder, elbow and wrist (Figure 8 and Table 5), as well as with enhanced rotational motion at the playing-side upper arm, shoulder line, and hip line (Figure 9 and Table 6). Conversely, ball speed decreased with excessive contralateral shoulder horizontal flexion/extension and playing-side elbow flexion-extension (Figure 10 and Table 7). These features, derived from 33 landmarks, 19 inter-keypoint segments, and 12 joint angles, comprehensively characterize forward stroke mechanics in table tennis. They reveal biomechanical principles for optimizing body segment activation to achieve peak ball speed.

Our findings align with prior studies (43, 44), confirming that playing-side arm linear velocity and positional range directly enhance ball speed (Figure 8). Racket speed, the direct determinant of ball speed, originates from the upper limb’s kinetic chain through sequential joint velocity propagation from shoulder to wrist (4, 45). The playing-side shoulder serves as the proximal driver, generating angular momentum that transmits distally to the elbow and wrist (45). These results support previous evidence linking playing-side shoulder motion to racket speed (4, 46, 47) (Figure 10, Table 7).

The contralateral shoulder showed negative correlations between horizontal flexion-extension range and velocity and ball speed. This suggests that minimizing non-playing-side arm motion relative to the torso improves stroke efficiency. Stabilizing the contralateral shoulder through scapular muscles anchors the upper arm during forehand strokes, enhancing whole-body power transfer and movement consistency.

Researchers disagree about elbow angular velocity. Xiao et al. reported positive correlations between elbow angular velocity and ball speed (44), while Zheng et al. found no significant correlation between playing-side elbow angular velocity and ball speed (43). Chen et al. found that elite players had smaller elbow flexion angles but greater elbow flexion angular velocities at impact (48). We found weak negative correlations between playing-side elbow angular range, angular velocities and ball speed (*r*_ws_ = −0.35 to −0.17) (Table 7, Figure 10). This difference may result from different motion phase divisions compared to other studies. Further experiments are needed to validate these findings.

Hip motion critically influences trunk rotation, which forms the foundation of kinetic chain initiation. Racket speed at impact was related to the hip axial rotation torque at the playing side (49). While previous studies established the importance of hip kinematics (1–3, 46), our analysis provides higher-resolution evidence that hip positional range and velocity (particularly along the x-axis) and inter-hip angular dynamics in the xz-plane positively correlate with ball speed (Figures 8, 9).

Force transmission begins with lower limb engagement, where playing-side leg activity (positive correlations) contrasts with contralateral leg stabilization (negative correlations) (Figures 8, 9). During forward swing, weight shifts toward the playing-side leg, positioning it closer to the rotational axis to bear load, while the contralateral leg balances and stabilizes rotation. Knee flexion-extension range and velocity negatively correlated with ball speed (Figure 10), indicating that minimizing knee movement during forward swing helps maintain efficient trunk rotation. Excessive knee motion appears to compromise this rotation, likely by introducing unnecessary vertical displacement that disrupts kinetic transfer.

Previous table tennis kinematic studies used keypoint positions and linear velocities (43), body segment angles and angular velocities (1, 3, 49, 50), and joint angles and angular velocities (1–3, 48, 50). These metrics included mean values (2), peak values (2, 48), and kinematic or racket movement characteristics at impact (2, 43, 48, 49). We comprehensively applied these metrics within the MediaPipe lightweight framework and provided more intuitive analysis of these kinematic features and their relationships with ball speed. Players with extensive professional training not only generate high-speed balls but also maintain excellent body movement stability and consistency (1, 3, 49, 50). This stability is crucial for continuous, stable, high-speed striking in high-level competition.

Beyond individual performance assessment, MediaPipe-based analysis enables population-level insights into developmental trends. Between-subject correlations reveal that female players’ forehand speed increases with age and height before 14.3 years but plateaus after 14.3 years (Table 8, Figure 11). This analysis provides valuable guidance for athletes at different developmental stages. For example, young players from pre-adolescence to early adolescence should balance fundamental technical training with strength and speed development to improve ball velocity and enhance attacking capabilities. In contrast, during middle to late adolescence, players must prioritize technical, tactical, psychological, and fitness factors over reliance on physical growth to advance performance.

### 4.2 MediaPipe-based table tennis analysis solution

MediaPipe is an open-source framework created by Google that provides cross-platform machine learning solutions for real-time perception tasks including human pose tracking, body keypoint detection, hand tracking, facial analysis, face detection, object detection, and augmented reality applications. The framework offers superior computational efficiency with lower latency and cross-platform compatibility across Linux, macOS, Windows, Android, and iOS platforms, making it highly suitable for practical applications (12). Its vision-based approach eliminates dependency on specialized hardware, enabling flexible deployment with consumer-grade cameras while maintaining computational efficiency. The system tracks 33 anatomical landmarks across consecutive frames to model temporal kinematics of human motion, effectively balancing accuracy with low computational overhead.

Researchers investigated MediaPipe’s reliability by comparing it with widely recognized accurate optoelectronic systems (e.g., VICON and Qualisys). Hii et al. used MediaPipe 3D for gait analysis and reported good to excellent agreement across spatiotemporal parameters, with good (ICC(2,1) >0.75) to excellent (ICC(2,1) >0.90) agreement in all temporal gait parameters except right-to-left leg transition time (ICC(2,1) >0.50), attributed to the very short duration (0.20 s) (51). Roggio et al. applied MediaPipe to obtain 3D joint angles (shoulder adduction, hip adduction) from 250 healthy volunteers, confirming high reliability of ML-driven posture analysis (ICC 0.67–0.95), with hip adduction showing the highest ICC (0.95) and knee valgus showing the lowest (0.67) (52). Latreche et al. compared 3D measurements with goniometer and digital inclinometer results, finding MediaPipe shoulder motion measurements all showed ICC >0.81: shoulder abduction ICC = 0.968, adduction = 0.99, extension = 0.99, flexion = 0.992, indicating excellent reliability. Mean differences were $-0.01^{\circ }$ compared to goniometer and $-0.36^{\circ }$ compared to digital inclinometer, with 95% limits of agreement confirming good validity (53).

Despite questions about MediaPipe 3D measurement accuracy, particularly at specific angles or during occlusion (13), MediaPipe 2D measurements have proven accurate and reliable (10, 54). Hamilton et al. compared MediaPipe 2D joint angles and range of motion with 3D motion capture systems (Qualisys), finding mean CV below 10% and CC = 0.95, demonstrating MediaPipe 2D accuracy (54). Some researchers compute 3D coordinates through post-processing of 2D measurements using multiple cameras (14, 55). Ceriola et al. used two cameras to acquire 2D keypoints and estimated 3D coordinates through stereo triangulation, reporting minimum absolute errors of ($3.1^{\circ }\pm 1.8^{\circ }$) and ($3.5^{\circ }\pm 1.9^{\circ }$) for hip joints and ($4.0^{\circ }\pm 3.7^{\circ }$) and ($4.8^{\circ }\pm 4.3^{\circ }$) for knee joints (55). We did not map MediaPipe’s camera-based 3D coordinates to anatomical coordinate systems but preserved the original coordinates. This approach retains MediaPipe’s relatively accurate x and y values, while z-axis depth variations do not affect accuracy in the plane perpendicular to the camera axis (xy-plane).

### 4.3 Limitations and future works

The study has several limitations. First, despite including 8–10 forehand strokes per player, only the fastest and slowest strokes were paired to calculate within-subject correlation coefficients. Elite participants exhibited highly consistent stroke patterns, leaving minimal variations in body motions and ball speeds. Measurement errors occasionally blurred speed distinctions, misclassifying fast strokes as slow and vice versa. Prioritizing extreme-speed strokes mitigated overlap effects but reduced statistical power. Two solutions could resolve this issue: (1) integrating high-speed cameras for precise measurements, albeit at the cost of practicality, or (2) recruiting lower-skilled players, who inherently display broader ball speed variations. Future work will refine the ball speed measurement model for higher precision, expand the participant pool to include diverse skill levels.

Second, this study recruited female provincial athletes, which limits the generalizability of findings to other populations. For example, male athletes may display different kinematic characteristics due to variations in movement patterns and skill levels. Future research should include mixed-gender cohorts or develop population-specific feature models for different demographics, including gender, age, and training level.

Real-time systems offer greater value for technical diagnosis. However, implementing real-time solutions requires addressing several technical challenges: (1) Action segmentation: Deep learning models must classify continuous time-series data into discrete stroke types (e.g., forehand strokes, backhand strokes, forehand chops, and backhand chops). (2) Success/failure classification: The system must distinguish successful shots from faults by analyzing ball trajectories and automatically detecting net contacts or boundary violations. (3) Automated ball speed measurement: Current ball trajectory calibration relies on manually annotated video coordinates. Real-time automation requires machine learning approaches, such as Ji et al.’s framework (56), which integrates VOCUS-based image segmentation, LGP+Adaboost classification for smear detection, and dynamic ROI optimization to address environmental noise, motion blur, and computational delays. (4) Accurate racket-ball impact timing is essential for movement phase segmentation. Machine learning models must automatically detect trajectory discontinuities to precisely calibrate impact moments based on ball flight path changes. (5) Player movement tracking: Players move rapidly during rallies, causing partial occlusion or frame exit. Wide-angle lenses expand the field of view, while advanced deep learning algorithms can reduce occlusion effects.

## 5 Conclusions

This study scanned 33 skeletal landmarks, 19 segments, and 12 joints using MediaPipe to identify kinematic features linked to ball speed in table tennis forehand strokes. These features may enable lightweight technical evaluation. Ball speed increased with greater playing-side arm linear movement at the shoulder, elbow and wrist, as well as with enhanced rotational motion at the playing-side upper arm, shoulder line, and hip line. Conversely, ball speed decreased with excessive contralateral shoulder horizontal flexion/extension and playing-side elbow flexion-extension. These kinematic patterns comprehensively characterize forward stroke mechanics, providing critical metrics for technical assessment and improvement. MediaPipe demonstrated robust performance, showing high consistency during repetitive motions. Its low-cost, cross-platform compatibility, high computational efficiency, minimal hardware dependency, and open-source nature position it as a promising tool for real-time biomechanical analysis in table tennis training systems.

## Data Availability

The datasets presented in this study can be found in online repositories. The names of the repository/repositories and accession number(s) can be found below: https://doi.org/10.6084/m9.figshare.28881086.
